# Entorhinal Cortex Functional Connectivity during Item Long-Term Memory and the Role of Sex

**DOI:** 10.3390/brainsci13030446

**Published:** 2023-03-04

**Authors:** Dylan S. Spets, Scott D. Slotnick

**Affiliations:** 1 Department of Psychiatry, Massachusetts General Hospital, Harvard Medical School, Boston, MA 02114, USA; 2 Department of Psychology and Neuroscience, Boston College, Chestnut Hill, MA 02467, USA

**Keywords:** sex differences, gender differences, gPPI, entorhinal cortex, perirhinal cortex

## Abstract

A growing body of literature shows there are sex differences in the patterns of brain activity during long-term memory. However, there is a paucity of evidence on sex differences in functional brain connectivity. We previously identified sex differences in the patterns of connections with the hippocampus, a medial temporal lobe (MTL) subregion, during spatial long-term memory. The perirhinal/entorhinal cortex, another MTL subregion, plays a critical role in item memory. In the current functional magnetic resonance imaging (fMRI) study, we investigated perirhinal/entorhinal functional connectivity and the role of sex during item memory. During the study phase, abstract shapes were presented to the left or right of fixation. During the test phase, abstract shapes were presented at fixation, and the participants classified each item as previously “old” or “new”. An entorhinal region of interest (ROI) was identified by contrasting item memory hits and misses. This ROI was connected to regions generally associated with visual memory, including the right inferior frontal gyrus (IFG) and visual-processing regions (the bilateral V1, bilateral cuneus, and left lingual gyrus). Males produced greater connectivity than females with the right IFG/insula and the right V1/bilateral cuneus. Broadly, these results contribute to a growing body of literature supporting sex differences in the brain.

## 1. Introduction

The MTL is critical for long-term memory and has specialized subregions—the perirhinal/entorhinal cortex, the parahippocampal cortex, and the hippocampus. According to the predominant model of MTL specialization, the perirhinal/entorhinal cortex processes item information, the parahippocampal cortex processes context information, and the hippocampus binds item and context information into a unified memory representation [1,2]. MTL subregions are among the most sexually dimorphic regions of the brain [3], and sex differences have been identified in MTL function [4,5,6]. Broadly, males have been found to activate MTL subregions, particularly the hippocampus and parahippocampal cortex, to a greater degree than females during long-term memory [6,7].

Although there is mounting evidence supporting sex differences in patterns of brain activity, sex differences in structural and functional brain connectivity are less well understood. Using diffusion tensor imaging, one study identified greater between-hemisphere (interhemispheric) connections in females compared with males and greater within-hemisphere (intrahemispheric) connections in males compared with females [8]. Resting-state studies have identified greater functional connectivity in sensorimotor, visual, and anterior lateral prefrontal regions for males than for females and greater functional connectivity within the default mode network for females than for males [9]. Very few studies have investigated sex differences in functional connectivity during long-term memory. In a previous investigation, we identified sex differences in the patterns of hippocampal connectivity [4]. Males exhibited greater hippocampal functional connectivity than females with the medial posterior frontal cortex, the anterior prefrontal cortex, the precuneus, and the cingulate sulcus. Mirroring the structural connectivity findings discussed above, we also found that females had a greater number of interhemispheric connections than males with the hippocampus, and males had a greater number of intrahemispheric connections than females with the hippocampus.

There is a growing body of literature demonstrating the importance of connectivity—particularly MTL connectivity—for maintaining memory function with age [10,11,12]. In light of this, and the evidence presented above, applying a sex-dependent lens to the functional connectivity studies of memory is critical for understanding the normal functioning of memory circuitry and the etiologies of memory-related diseases. The MTL is one of the first regions targeted in normal and pathological aging [13]. These regions are among the first in the brain to accumulate amyloid and tau proteins that are hallmark pathologies of Alzheimer’s disease (AD) [14,15,16]. Moreover, there is a sex difference in the prevalence of memory-related disorders, including AD, with a greater incidence in females than males [17].

**Figure 1:**
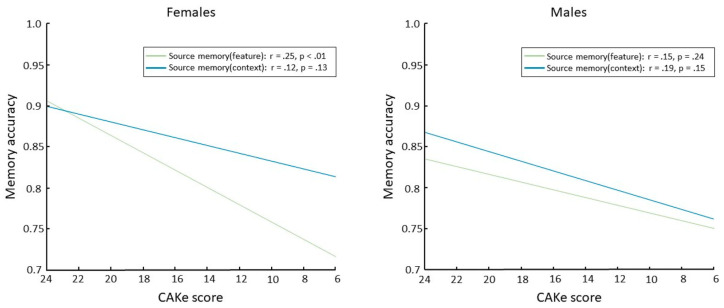
Source memory accuracies as a function of CAKE scores for females (**left**) and males ((**right**); correlation values and *p*-values at the upper right of each panel). Reprinted from *Brain and Cognition*, 166, Haley A. Fritch, Lauren R. Moo, Madeline A. Sullivan, Preston P. Thakral, and Scott D. Slotnick, “Impaired Cognitive Performance in Older Adults is Associated with Deficits in Item Memory and Memory for Object Features”, 105,957, copyright (2023), with permission from Elsevier.

In an effort to increase the accessibility and efficiency of cognitive assessments, we developed a novel self-administered global cognitive assessment tool, the cognitive assessment via keyboard (CAKe). Using the CAKe, we recently identified a sex-dependent deficit in source memory for item features associated with impaired cognitive performance [18]. Specifically, older female participants (aged 55–77) who scored lower on the CAKe (indicating cognitive impairment) had a deficit in source memory for item features (i.e., object color) but no significant deficit in source memory for item context (i.e., background color; Figure 1 left). Differential source memory effects were not observed in older male participants (Figure 1 right). These results suggest a female-specific impairment in source memory for features that is associated with cognitive impairment and may be explained by sex differences in MTL functioning. These findings are consistent with previous studies that have linked the perirhinal/entorhinal cortex with source memory for item features [19,20,21]. Considering these converging lines of evidence, examining sex differences in perirhinal/entorhinal connectivity should provide a better understanding of healthy and diseased aging of memory circuitry.

**Figure 2:**
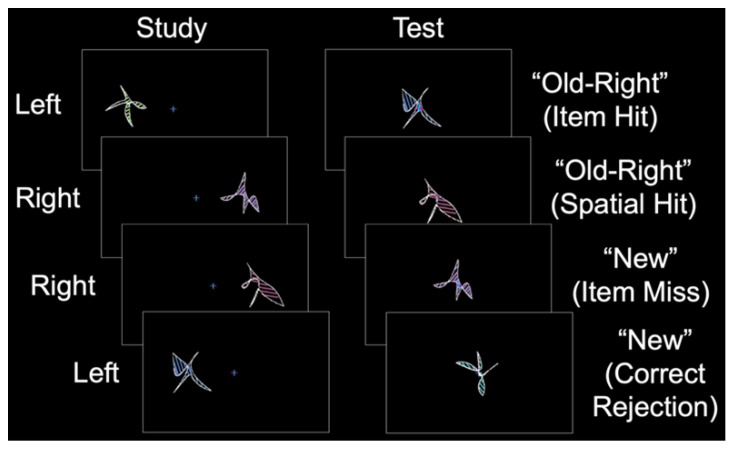
Behavioral protocol. During the study phase, abstract shapes were presented in the left or right visual field (labeled to the left). During the test phase, old and new shapes were presented at the center, and participants made “left”, “right”, or “new” judgments (illustrative responses shown to the right with corresponding response types in parentheses).

In the current fMRI study, we aimed to identify whether sex differences exist in the patterns of perirhinal/entorhinal connectivity during item long-term memory, a cognitive process known to activate this MTL subregion [1,2]. During the study phase, abstract shapes were presented in the left or right visual field (Figure 2). During the test phase, old and new shapes were presented at the center, and the participants made “left”, “right”, or “new” judgments. Item memory was isolated by contrasting “old” responses to old items (with incorrect spatial memory judgments; item memory hits) and “new” responses to old items (item memory misses) [22]. Critically, females and males were matched on item memory performance such that any differences between the groups could not be attributed to differences in behavioral performance. We predicted that during item memory, the perirhinal/entorhinal cortex would be connected to a broad set of memory regions including the prefrontal, parietal, and visual-processing cortices. Moreover, based on our recent finding of the relatively spared performance of cognitively impaired older males on source memory for item features [18], we predicted that males would have greater connectivity between the perirhinal/entorhinal cortex and other brain regions associated with memory.

## 2. Materials and Methods

### 2.1. Participants

In the present study, we reanalyzed data from two of our previous experiments [23,24], referred to as Experiment 1 [23] and Experiment 2 [24] below. Unless otherwise stated, the stimulus, data acquisition, and data analysis protocols in this paper were identical to our previous paper in which a general linear model (GLM) analysis was used to investigate sex differences in item memory, and the details of each experiment were identical [7]. Across both experiments, there were 15 female participants and 11 male participants. For the previous and current study, 11 females were selected from all the female participants to best match the item memory accuracy of the 11 male participants (an iterative matching procedure was used; see [7] for details).

### 2.2. Stimulus Protocol and Task

Participants completed a behavioral training session to familiarize themselves with the task. During fMRI, each participant completed a single anatomic scan and three or six study-test runs in Experiment 1 and Experiment 2, respectively. During each study phase, abstract shapes were presented to the left or right of fixation (Figure 2, left). During the corresponding test phase, shapes were presented at fixation for 2.5 s with a 4 to 12 s intertrial interval. The participants were instructed to remember each shape and its spatial location while maintaining fixation. During the test phase, the participants classified each shape as “old–left”, “old–right”, or “new”, followed by an “unsure” or “sure” confidence response with a button box that was placed in their left hand (Figure 2, right). In Experiment 1, the study phase consisted of 144 shapes, and the test phase consisted of 96 shapes (32 new shapes, 32 similar shapes, and 32 new shapes). In Experiment 2, the study phase consisted of 32 shapes, and the test phase consisted of 48 shapes (32 old shapes and 16 new shapes). Shape sets were pseudorandomized (with no more than 3 shapes of a given type presented sequentially), counterbalanced across the participants using a Latin square design, and never repeated across runs. Specific details on the stimulus protocol and task were reported in our previous item memory study [7].

### 2.3. Experimental Design

Item memory accuracy was the weighted percentage of accurately identified old and new shapes: p (old) × old-hit rate + p (new) × new-correct rejection rate [25]. For each experiment, a subset of female participants was selected such that their mean item memory accuracy and variance best matched these behavioral metrics for the male participants [7].

### 2.4. Image Acquisition and Analysis

Images were acquired using a 3-Tesla Siemens Allegra MRI scanner. Anatomic data were acquired using a multiplanar rapidly acquired gradient echo (MPRAGE) sequence (TR = 30 ms, TE = 3.3 ms, 128 slices, 1 × 1 × 1.33 mm resolution). Functional data were acquired using a T2*-weighted echo planar imaging sequence (TR = 2000 ms, TE = 30 ms, 64 × 64 acquisition matrix, 26 to 30 slices in Experiment 1 and 30 slices in Experiment 2).

A random-effect GLM analysis was conducted in SPM12 (Wellcome Trust Center for Neuroimaging, London, UK). Functional image preprocessing included slice-time correction, motion correction (to the first volume of each run), and spatial normalization to the Montreal Neurological (MNI) template (which included 2 mm^3^ resampling). The GLM included 18 motion regressions (3 translation and 3 rotation parameters along with their first- and second-order derivatives) and scrubbing of all data with a framewise displacement of ±0.5 mm [26]. We previously employed the same motion correction pipeline to investigate hippocampal connectivity during spatial memory [4]. To maximize spatial resolution, spatial smoothing was not conducted. Anatomic images were normalized to MNI space (with 1 mm^3^ resolution) and averaged across the participants. Item memory was isolated by contrasting accurate item recognition with inaccurate spatial identification (i.e., “left”/right responses, “left” response to those items previously presented on the right, or “right”/left responses) and item misses (i.e., “new”/right or “new”/left). Confidence responses were collapsed for all analyses.

The contrast of item hits versus misses (inclusive of all participants) was thresholded at *p* < 0.01, uncorrected, to define a perirhinal/entorhinal cortex ROI for the functional connectivity analysis. This contrast produced a single activation in the entorhinal cortex. A 3 mm radius sphere was extracted around the activation, and this was used as the perirhinal/entorhinal cortex ROI. A 3 mm sphere was chosen as it most effectively encompassed the ROI, allowing for the sampling of a sufficient number of voxels without sampling from the surrounding regions of no interest or white matter. The voxels contained in this sphere were used as a seed for the functional connectivity analysis. The uncorrected contrast also produced activity in other canonical memory regions, including the hippocampus. We have previously identified sex differences in the brain during item memory using a GLM approach and the contrast, corrected for multiple comparisons, identified many long-term memory regions [7]. We have employed similar methods to investigate sex differences in the functional connectivity of the hippocampus [4] and thalamus [27] during spatial long-term memory. Importantly, in addition to investigating a different memory type in the current study (i.e., spatial memory versus item memory), the ROIs employed in these previous studies were completely distinct from the present ROI.

Functional connectivity analyses were conducted using a generalized psychophysiological interaction (gPPI) toolbox [28] using the individual-participant first-level models of item memory hits versus misses. For each participant, whole-brain *t*-contrasts of entorhinal functional connectivity were created, and a random-effect analysis was conducted to determine the voxels functionally connected to the entorhinal cortex.

All contrasts were thresholded at *p* < 0.01, and cluster-extent-corrected to *p* < 0.05 (except for the contrast to identify the entorhinal cortex ROI). To compute the cluster-extent threshold, we first estimated the spatial autocorrelation for the gPPI contrast of item memory hits versus misses (across all participants) using a custom script (img_xcorr.m; Slotnick, n.d., obtained from https://sites.google.com/bc.edu/sd-slotnick/publications/scripts-and-stimuli (accessed on 1 February 2023)) and employed the smallest spatial autocorrelation value (3.75 mm) across the experiments (larger spatial autocorrelations can be assumed to be due to true activations rather than noise). Ten thousand Monte Carlo simulations were conducted based on the acquisition volume parameters, estimated spatial autocorrelation, and the desired individual voxel and family *p*-value [29]. This resulted in a cluster extent of 46 voxels that was applied to all the contrasts. Images were imported into MRIcroGL (obtained from www.nitrc.org (accessed on 3 February 2023)) and overlaid on the average anatomic for viewing purposes.

Beta-weights were extracted from the activations of interest produced by the group functional connectivity map of item hits versus misses using the REX toolbox [30] (obtained from https://web.mit.edu/swg/software.htm (accessed on 10 February 2023)) and imported into the Statistical Package for Social Science (SPSS) for further analyses. For each participant and ROI, beta-weights were entered into a GLM to examine the role of sex. One female participant was excluded from these analyses based on connectivity magnitudes that were more than two standard deviations above the mean (i.e., 11 males and 10 female participants were included in the final models). Sex differences have been shown to exist in the rate of maturation of brain regions, including a subset of those that were included as ROIs in these analyses and within the age range of this sample [31,32,33]. As such, age was entered as a covariate in the model to control for possible confounding effects.

## 3. Results

Across all the participants (15 females, 11 males), there was no significant difference in item memory accuracy between females (68.32 ± 1.29%, mean ± standard error, chance = 50%) and males (66.59 ± 1.79%, weighted F(1,24) < 1). For the fMRI analysis, we selected 11 female participants such that behavioral accuracy and variance was even better matched to the males (female performance = 66.86 ± 1.41%, t(20) < 1).

**Figure 3:**
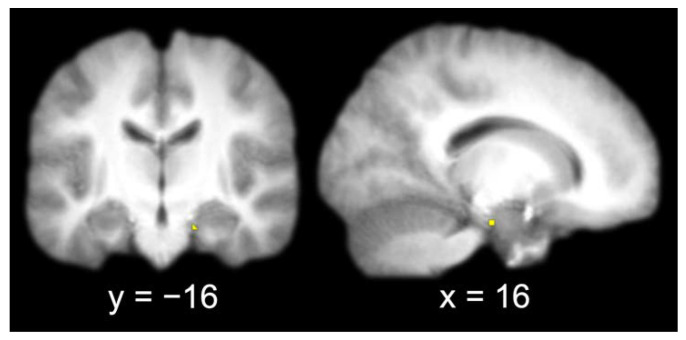
Entorhinal cortex seed (identified by contrasting item memory hits and misses and extracting a 3 mm radius sphere around the most significant coordinate of the activation; left, coronal slice; right, sagittal slice).

The group contrast of item hits versus misses produced a single activation in the right entorhinal cortex (x = 16, y = −16, z = −22). A 3 mm sphere centered at the most significant coordinate contained 6 voxels that were used as the seed for the functional connectivity analysis (Figure 3).

**Figure 4:**
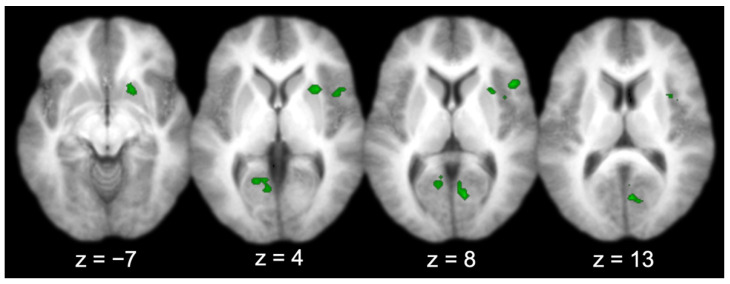
Entorhinal cortex functional connectivity group results (for all participants; axial slices).

Across all the participants, there were several regions functionally connected to the entorhinal cortex (Table 1 and Figure 4), including the prefrontal cortex (right IFG; middle two panels, top right lateral), visual-processing regions (bilateral V1, bilateral cuneus, and left lingual gyrus; right three panels, bottom), the right insula (right three panels, top right lateral–medial), and the right putamen (left three panels, top right medial). Three activations produced by the group connectivity analysis (i.e., the right IFG/insula, the right V1/bilateral cuneus, and the left V1/lingual gyrus) were selected to test the effect of sex, based on previous findings [5,6,7].

**Figure 5:**
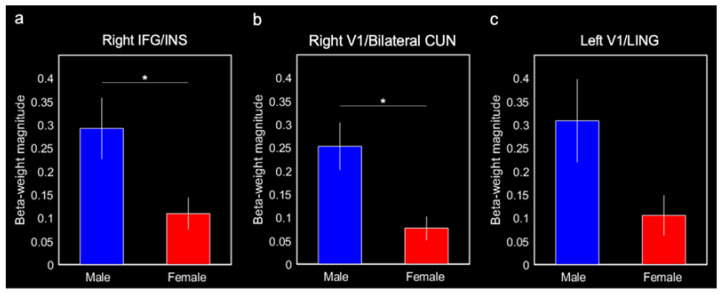
Mean beta-weight magnitudes extracted from three ROIs identified in the group entorhinal cortex connectivity results for males and females. Mean beta-weight magnitudes represent connectivity between the entorhinal cortex and (**a**) right IFG and insula (INS), (**b**) right V1 and cuneus (CUN), and (**c**) left V1 and lingual gyrus (LING). Error bars represent ± 1 standard error of the mean. * Bonferroni-corrected *p* < 0.05.

Males produced greater connectivity between the entorhinal cortex and the right IFG/insula (0.29 ± 0.07, F(1,20) = 8.86, *p* = 0.010, Bonferroni-corrected *p* < 0.05) and the right V1/bilateral cuneus (0.25 ± 0.05, F(1,20) = 10.38, *p* = 0.0047, Bonferroni-corrected *p* < 0.05) compared with females (0.11 ± 0.03 and 0.08 ± 0.03, respectively; Figure 5a,b). Males also produced greater connectivity between the entorhinal cortex and left V1/lingual gyrus (0.31 ± 0.09) compared with females (0.11 ± 0.04; F(1,20) = 5.03, *p* = 0.038; Figure 5c); however, this effect was not corrected for multiple comparisons (Bonferroni-corrected *p* > 0.1). Although they were not a priori ROIs, we ran post hoc comparisons on the putamen and putamen/insula activations for completeness. There were no significant sex differences in connectivity with these activations (both *p*-values > 0.2).

## 4. Discussion

To our knowledge, this is the first study to examine perirhinal/entorhinal cortex connectivity as a function of sex. Compared with females, males had greater connectivity between the perirhinal/entorhinal cortex and the right IFG/insula as well as the right V1/bilateral cuneus. There were no regions that exhibited greater connectivity with the perirhinal/entorhinal cortex for females relative to males. These results support our hypothesis that decreased connectivity with the perirhinal/entorhinal cortex in females may explain the deficit in source memory for item features that were observed in cognitively impaired females, but not males [18]. Future studies of aging should investigate perirhinal/entorhinal cortex connectivity and associations with source memory for item features to better understand how these connections may impact memory function with age.

Compared with females, males produced greater connectivity between the perirhinal/entorhinal cortex and left V1. Although males produced greater connectivity with right V1, this difference was not corrected for multiple comparisons. These results suggest that there may be differences in the hemispheric lateralization of perirhinal/entorhinal connectivity. There are several hypotheses that may explain this hemispheric difference. First, male brains are structurally optimized for intrahemispheric connectivity [8]. As the perirhinal cortex seed was in the right hemisphere, the intrahemispheric connectivity hypothesis would posit that men would produce greater connectivity with brain regions in the right hemisphere (e.g., right V1). This hypothesis is also supported by the finding in the right IFG/insula (where men again produced greater connectivity relative to females). Second, there is a body of literature showing a female advantage in the types of memory in which verbal strategies can be employed and a male advance in the types of memory in which spatial strategies can be employed [34]. As the left hemisphere is specialized for verbal processing [35], and the right hemisphere is specialized for spatial processing [36], increased male connectivity in the right hemisphere may suggest that males utilize a spatial-based memory strategy in the current task, such as focusing on the spatial relationships between different item features. Future studies should further investigate sex differences in the hemispheric lateralization of MTL connections.

In the present study, we found that the perirhinal/entorhinal cortex was connected to several regions including the prefrontal cortex, visual-processing regions, the right insula, and the right putamen. As the perirhinal/entorhinal cortex passes item/context information onto the hippocampus, the predominant models of MTL specialization [1,2] would predict these regions to be functionally connected. However, we did not observe perirhinal/entorhinal connectivity with the hippocampus. An alternative model of perirhinal/entorhinal connectivity posits that the perirhinal/entorhinal cortex plays a role in the retrieval of memories that have been transferred to the cortex [37]. The perirhinal/entorhinal cortex’s role in the retrieval of neocortical memory representations is supported by anatomic connectivity, as this region shares reciprocal connections with the cortex [38]. Moreover, studies in primates suggest that the perirhinal/entorhinal cortices receive previously encoded features of memories (e.g., visual representations of objects) and simultaneously retrieve the associated features of these objects to retrieve a full memory representation [39]. The perirhinal/entorhinal cortex has also been identified as part of the rapid visual representation system, which also includes the frontal and parietal regions. According to this model, this set of brain regions rapidly recognizes previously presented objects or items while bypassing the hippocampus [40]. Critically, these models of perirhinal/entorhinal connectivity and function are not mutually exclusive. Although the current set of results supports the second and third models of perirhinal/entorhinal function, the null findings of connectivity between the perirhinal/entorhinal cortex and the hippocampus could be due to the relatively large cluster-extent threshold employed, a lack of power, the nature of the task, or the specific contrast employed.

## 5. Conclusions

There is substantial evidence that there are sex differences in the brain during long-term memory [6]. The current results extend these findings by showing that perirhinal/entorhinal cortex connectivity differs between females and males during long-term item memory. These results are consistent with previous findings illustrating a female-specific deficit in source memory for item features associated with cognitive impairment [18]. Examining sex differences in perirhinal/entorhinal cortex connectivity in studies of aging may ultimately help inform our understanding of how memory circuitry and function are altered with age. Broadly, the current and previous findings argue against the common practice of collapsing across sex in cognitive neuroscience studies.

## Figures and Tables

**Table 1:** Regions functionally connected to the entorhinal cortex during item memory for all participants.

Region	BA	x	y	z	k
R. IFG/Insula	44	47	18	8	75
R. V1/Bilateral Cuneus	17/18	5	−68	13	107
L. V1/Lingual Gyrus	17/18	−10	−58	7	49
R. Putamen/Insula	−	28	13	4	53
R. Putamen	−	19	13	−7	47

BA refers to the Brodmann area, MNI coordinate (x, y, z) refers to the center of each activation, and k refers to the number of voxels in each cluster.

## Data Availability

The data presented in this study are available on request from the corresponding author.
